# *Drosophila* Larvae-Inspired Soft Crawling Robot with Multimodal Locomotion and Versatile Applications

**DOI:** 10.34133/research.0357

**Published:** 2024-05-03

**Authors:** Qin Fang, Jingyu Zhang, Yinhui He, Nenggan Zheng, Yue Wang, Rong Xiong, Zhefeng Gong, Haojian Lu

**Affiliations:** ^1^ State Key Laboratory of Industrial Control and Technology, Zhejiang University, Hangzhou 310027, China.; ^2^ Institute of Cyber-Systems and Control, Zhejiang University, Hangzhou 310027, China.; ^3^ Medical School of Zhejiang University, Hangzhou 310027, China.; ^4^ College of Computer Science and Technology, Zhejiang University, Hangzhou 310027, China.; ^5^ Stomatology Hospital, School of Stomatology, Zhejiang University School of Medicine, Zhejiang Provincial Clinical Research Center for Oral Diseases, Key Laboratory of Oral Biomedical Research of Zhejiang Province, Cancer Center of Zhejiang University, Engineering Research Center of Oral Biomaterials and Devices of Zhejiang Province, Hangzhou 310000, China.

## Abstract

Soft crawling robots have been widely studied and applied because of their excellent environmental adaptability and flexible movement. However, most existing soft crawling robots typically exhibit a single-motion mode and lack diverse capabilities. Inspired by *Drosophila* larvae, this paper proposes a compact soft crawling robot (weight, 13 g; length, 165 mm; diameter, 35 mm) with multimodal locomotion (forward, turning, rolling, and twisting). Each robot module uses 4 sets of high-power-density shape memory alloy actuators, endowing it with 4 degrees of motion freedom. We analyze the mechanical characteristics of the robot modules through experiments and simulation analysis. The plug-and-play modules can be quickly assembled to meet different motion and task requirements. The soft crawling robot can be remotely operated with an external controller, showcasing multimodal motion on various material surfaces. In a narrow maze, the robot demonstrates agile movement and effective maneuvering around obstacles. In addition, leveraging the inherent bistable characteristics of the robot modules, we used the robot modules as anchoring units and installed a microcamera on the robot’s head for pipeline detection. The robot completed the inspection in horizontal, vertical, curved, and branched pipelines, adjusted the camera view, and twisted a valve in the pipeline for the first time. Our research highlights the robot’s superior locomotion and application capabilities, providing an innovative strategy for the development of lightweight, compact, and multifunctional soft crawling robots.

## Introduction

Soft crawling robots [1,2], typically composed of materials with a low Young’s modulus like silicone rubber, are actuated by intelligent materials such as dielectric elastomer [3], shape memory alloy (SMA) [4], magnetically active elastomer [5–7], liquid crystal elasticity [8], piezoelectric material [9], ionic polymer metal composite [9], etc. Their inherent flexibility enables passive deformation, facilitating adaptation to diverse terrains and environments. Furthermore, in narrow and complex settings, these robots demonstrate agile obstacle avoidance and less prone to substantial damage during collisions [10,11]. Consequently, soft crawling robots hold considerable promise across diverse applications [12–14], including exploring unknown environments, executing rescue missions, and conducting pipeline exploration.

Drawing inspiration from nature has always been an important design approach in the field of soft crawling robots. On the basis of different crawling mechanisms, soft crawling robots can be categorized into legged robots [15,16], inchworm-like robots [17,18], snake-like robots [19,20], and caterpillar-like robots [8,21]. Legged robots move by sequentially grounding their legs, offering advantages in speed but suffering from complexity in structure, and their slender limbs are susceptible to getting stuck in terrains with grooves and cracks. Inchworm-like robots achieve locomotion by alternately anchoring their front and rear feet, combined with the bending and straightening of their torso. This crawling mechanism simplifies the design and control of the robot, but its reliance on substantial body deformation limits its capability in constrained environments. Unlike inchworm-like robots that create longitudinal bending, snake-like robots move by generating lateral bending, producing continuous transverse waves for locomotion. Similarly, this mode of movement is also limited in extremely narrow spaces. Caterpillar-like robots adopt peristaltic locomotion, moving forward through alternating expansion and contraction of their body segments. This movement is slower but offers the advantages of stability, lower energy consumption, and high adaptability to various terrains. Das et al. [22] designed a robot with 5 segments, each comprising a single air chamber, enabling forward movement. Robertson and Paik [23] developed a multisegment robot, each segment featuring 3 air chambers, capable of peristaltic advancement, turning, and even rolling. Liu et al. [24] designed a pipeline inspection robot, combining steering modules, elongation modules, and anchoring modules for navigating and actively turning in Y-shaped pipelines. However, these robots, primarily based on bending and extension actuators, typically lack twisting capacity, posing challenges in adjusting their viewing angles and operational orientations in constrained environments.

To address the limitations in robotic torsional capabilities, researchers have developed various flexible twisting actuators. Many researchers used a combination of helical strain-limiting structures and flexible expansion mechanisms. Choi et al. [25] reported an inflatable twisting actuator that achieves a torsion of up to 600° while avoiding bulking through a double-helix tendon routing path. With the rising interest in origami mechanisms in robotics, particularly the development of twisting actuators inspired by the Kresling pattern [26,27], a significant solution has been provided. Li et al. [28] proposed a silicone-based twisting actuator, combining multiple actuators with varying twisting orientations, effectively positioning the grasped object and adjusting the camera’s perspective in pipelines. However, existing twisting actuators often twist only in one direction, and few robots possess capabilities for forward movement, turning, rolling, and twisting simultaneously. Designing a soft crawling robot with multimodal locomotion, especially one that includes twisting capabilities, remains a challenge.

In nature, *Drosophila* larvae have a compact lightweight body structure and can achieve a variety of flexible movements such as moving forward and backward, turning, rolling, twisting, raising their head, flicking their tails, etc., which have attracted widespread attention from researchers [29,30]. Techniques like noninvasive calcium imaging [31] have been instrumental in precisely observing and studying the larva’s muscular activity and movement patterns, thereby offering significant inspiration for soft crawling robot design. The muscles of *Drosophila* larvae are mainly divided into transverse, longitudinal, and oblique groups, with the oblique muscles being predominant. The synergistic functionality of these oblique muscles lays a foundational role in facilitating the larva’s versatile movement capabilities.

Inspired by *Drosophila* larvae, we propose a novel multimodule soft crawling robot capable of multiple modes of movement. Each module is equipped with 4 obliquely positioned SMA coils, enabling shrinkage (60% shrinkage), bending (39°), and torsion (43°). We analyzed the mechanical properties of the robot modules through experiments and finite element simulations and evaluated their movement speed on various surfaces and at different control frequencies. The robot, operated remotely via a handle, adeptly navigated “Z”-, “J”-, and “U”-shaped paths and a narrow maze, showcasing its obstacle avoidance capabilities. Finally, utilizing the robot module’s inherent bistable properties as an anchoring unit, the robot was used for pipeline application experiments. The robot efficiently performed inspections in horizontal, vertical, and curved pipelines, navigated branched pipelines with active steering, and demonstrated camera view adjustment and valve rotation abilities leveraging its torsional capability.

## Results

### Bioinspired design principles

**Fig. 1.**
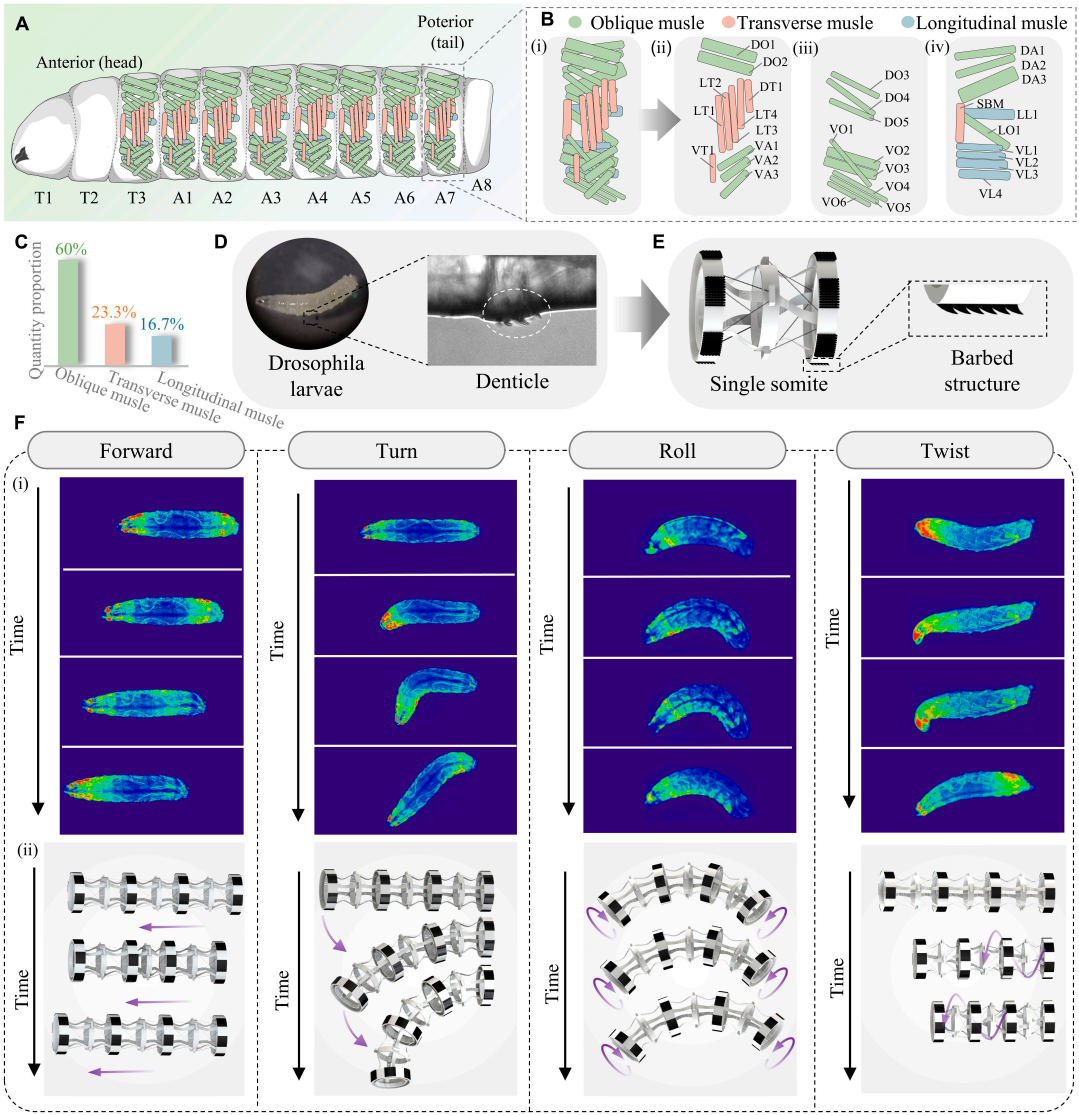
Bionics design basis for soft crawling robots inspired by *Drosophila* larvae. (A) Schematic diagram of the anatomy of *Drosophila* larvae. LT, lateral transverse; VT, ventral transverse; DO, dorsal oblique; DT, dorsal transverse; LT, lateral transverse; VA, ventral acute; VO, ventral oblique; DO, dorsal oblique; DA, dorsal acute; SBM, segment border muscle; LL, lateral longitudinal; LO, lateral oblique; VL, ventral longitudinal. (B) Muscle distribution in single somite where oblique muscles, transverse muscles, and longitudinal muscles are depicted in light green, light red, and light blue. (C) The proportion of each muscle type. Oblique muscles are the most prevalent at 60%, followed by transverse muscles at 23.3%, and longitudinal muscles at 16.7%. (D) Denticles below abdomen of *Drosophila* larvae to assist movement. (E) The structure drawing of robot’s body module with the barbed structure that inspired by denticles. (F) The muscular activity of *Drosophila* larvae in various movement modes (i) and corresponding multimodal locomotion of the proposed robot (ii).

This section presents the design methodology for a soft crawling robot inspired by *Drosophila* larvae. Initially, we introduce an anatomical and muscular distribution diagram of the *Drosophila* larva to illustrate the biomimetic foundation of the robot design. As shown in Fig. 1A, the larva’s body comprises multiple body segments, typically including 3 thoracic segments (T1 to T3) and 8 abdominal segments (A1 to A8). The muscular structure within these body segments is generally similar [shown in Fig. 1B (i)], and according to their proximity to the larva’s body center, they can be categorized into 3 layers. The innermost layer is represented in Fig. 1B (ii), and the outermost layer is in Fig. 1B (iv), with each muscle labeled accordingly. Muscles are generally classified as oblique muscles (depicted in light green), transverse muscles (shown in light red), and longitudinal muscles (in light blue). We quantified the proportion of each muscle type (Fig. 1C), finding oblique muscles to be the most prevalent at 60%, followed by transverse muscles at 23.3% and longitudinal muscles at 16.7%. The modular body structure and the muscular system of the larvae significantly enhance movement flexibility and adaptability, providing essential biomechanical principles and inspiration for designing soft crawling robots.

*Drosophila* larvae have many small denticles below their abdomen to assist movement, as illustrated in Fig. 1D. Inspired by the structure of denticles, we adopted barbed structures on the robot’s segments to improve locomotion efficiency (Fig. 1E). Six evenly distributed barbed structures were designed on each end of the robot’s segments, providing sufficient friction while minimizing the robot’s weight. Using fluorescence imaging technology, we analyzed the muscular activity of *Drosophila* larvae in various movement modes, as depicted in Fig. 1F (i). The coordinated interaction of the muscular system in *Drosophila* larvae contributes significantly to their agile movement, with the oblique muscles showing substantial activation in all movement modes.

In general, the integration of transverse, longitudinal, and oblique muscles enables robots to perform movements with greater precision and flexibility. However, in practical robot design, fully replicating the anatomy of *Drosophila* larvae would significantly increase the complexity of the robot’s electrical wiring and control systems, rendering it impractical. Considering the added redundancy and complexity in robot control with an increased number of muscles, we adopted a 4-muscle antagonistic configuration to design the robot module. This arrangement of oblique muscles generates driving forces in various directions, allowing for the robot module’s contraction, omnidirectional bending, and bidirectional twisting. Multiple robot modules are assembled to form a robot to achieve various motion modes. Figure 1F (ii) shows the state of the proposed robot during multimodal motion processes such as forward, bending, rolling, and twisting.

### Fabrication and characterization analysis

**Fig. 2.**
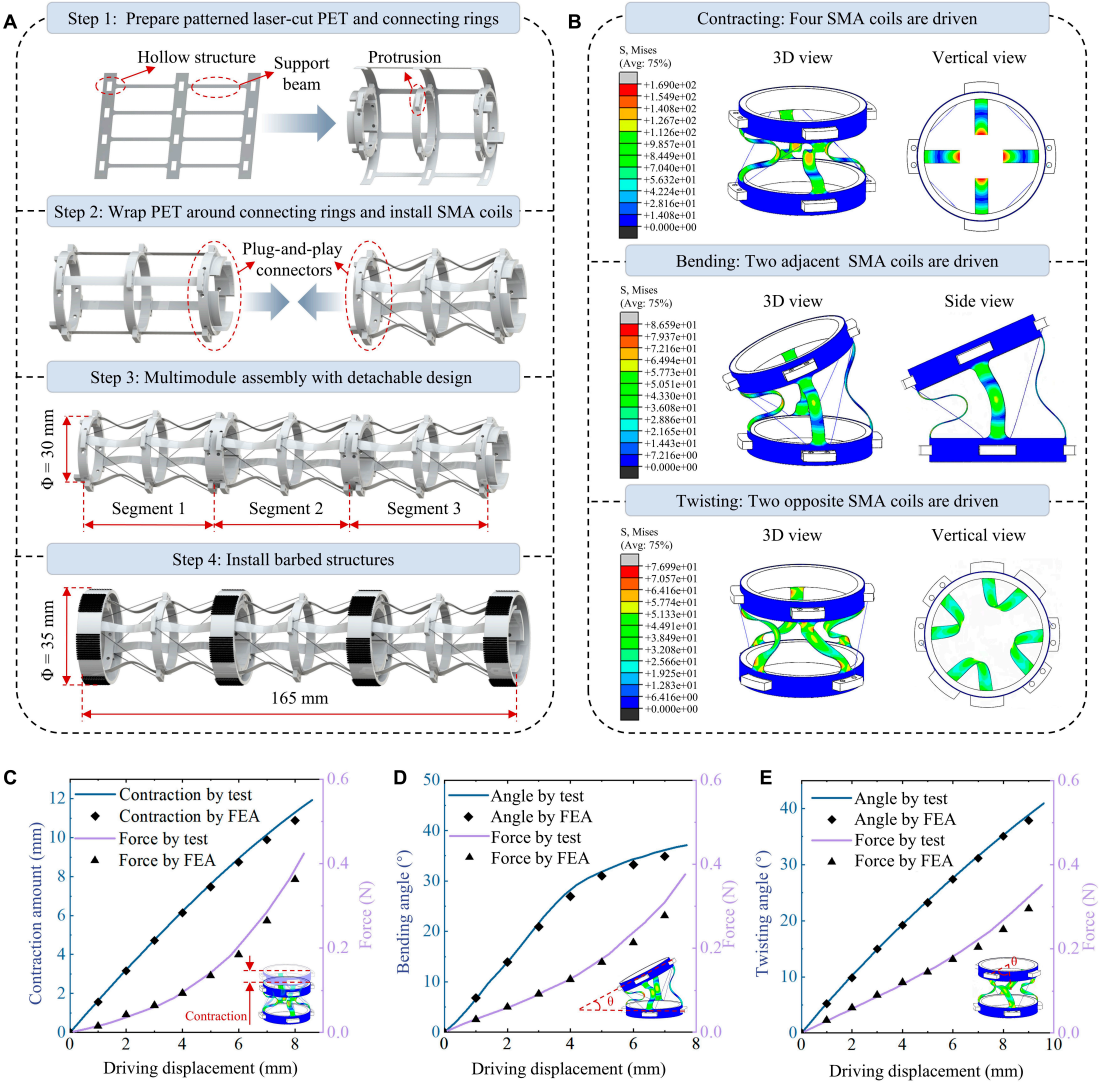
Fabrication and characterization of the proposed soft crawling robot inspired by *Drosophila* larvae. (A) Design and fabrication of the soft crawling robot. Initially, the support sheet made of PET material was cut into a specific shape and wrapped around the connecting ring, which were acted as plug-and-play connectors. Subsequently, 4 SMA coils were installed on the robot modules arranged in a diagonal configuration. Finally, barbed structures were installed at both ends of the robot segments, thus completing the construction of the soft crawling robot. (B) Finite element simulation analysis of robot module including contracting, bending, and twisting deformation. (C) Force and contracting amount curves of robot module with the increase in driving displacement. (D) Force and bending angle curves of robot module with the increase in driving displacement. (E) Force and twisting angle curves of robot module with the increase in driving displacement.

As mentioned in the previous section, *Drosophila* larvae have multiple body segments, which can be easily extended to modular robot design methods. The robot proposed in this study consists of 3 segments, each including 2 robot modules powered by the same driving sources. Figure 2A provided a detailed description of the manufacturing process for the soft crawling robot. Initially, the support sheet for each segment [made of polyethylene terephthalate (PET) material] was cut into a specific shape using a laser cutter. These sheets mainly included hollow structures and supporting beams, with the beams measuring 3 mm in width and 0.17 mm in thickness. The supporting sheet was wrapped around the connecting ring, aligning the ring’s protrusions with the hollow structures on the sheet to achieve accurate positioning. Then, the glue was used to bond the ends of the PET sheet. At this point, the elastic thin sheet and the connecting rings were firmly connected. Each pair of connecting rings acted as plug-and-play connectors, positioned at both ends of each robotic segment. The connectors were designed with a 1.5° inclination angle to facilitate the rapid assembly and disassembly of robot segments through interference fit, as shown in Fig. S1A and B. In cases where certain segments were damaged, they can be conveniently replaced, while also allowing robots to have different numbers of segments according to their usage scenarios. To assess the reliability of the connectors, this study tested the tensile force required to separate each pair of connectors, revealing that a force of 11.5 N is needed, which is 88 times the weight of the robot itself, as shown in Fig. S1C and D.

Subsequently, SMA coils needed to be installed on the robot modules. Each connecting ring’s protrusion was designed with installation holes, and the prepared SMA coils were mounted through these holes. The cold-pressed terminal was used to connect the SMA coil to the conducting thin wire (Fig. S3). The 4 SMA coils were arranged in a diagonal configuration, with the inclination directions of adjacent SMA coils being opposite. Since the length of the SMA coils was shorter than the distance between the installation holes, the support beams on the robot segments bent inward after the SMA coils installation. It is worth noting that the robot modules possess bistable characteristics, enabling the support beams to bend both inward and outward, a feature that will demonstrate its advantages in subsequent application experiments. Finally, barbed structures were installed at both ends of the robot segments, thus completing the construction of the soft crawling robot, which has a diameter of 35 mm and a length of 165 mm.

Finite element simulation analysis (Fig. 2B) and kinematic model (Fig. S3) were conducted to investigate the deformation ability of each module of the robot. When the 4 SMA coils contracted simultaneously, the 4 supporting beams further bent inward, resulting in a reduction in the height of the robot module, and the most significant stress concentration was observed in the midsection of the support beams. When adjacent SMA coils were activated to contract, the support beam located between the coils experienced augmented bending, while the support beam on the opposite side tended to straighten. This asymmetrical response resulted in the robot module undergoing a bending deformation, with the maximum stress occurring in the middle of the beam that bends the most. It is important to note that combining the 4 SMA coils allows the module to achieve omnidirectional bending. For simplicity in control, all bending deformations were achieved by driving 2 adjacent SMA coils in this study. The module would twist when one set of opposed SMA coils contracted. From a top view, it revealed a clockwise rotation (see Fig. 2B). If another set of SMA coils contracted, the module would twist counterclockwise. The maximum stress during twisting occurred at the intersection of the support film and the connecting ring, necessitating the design of rounded corners to reduce stress concentration in these areas.

In addition, characterization experiments of the robot module were conducted. As depicted in Fig. S4, a tensile machine was used to stretch the SMA coil, measuring both the elongation and the tensile force. Then, the robot module was mounted on the tensile machine and the clamping jaw pulling the tendon to simulate the contraction of the SMA coil. By the results shown in Fig. 2C, with the increase in driving displacement, the height of the module decreased linearly, and the force increased parabolically. The height of the robot module can be reduced by 12 mm from an initial height of 20 mm, achieving a contraction rate of 60% (Fig. S5A). Similarly, the characterization results of bending deformation are shown in the Fig. 2D. As the driving displacement of the SMA coil increased, the increase in bending angle gradually slowed down, while the tensile force significantly increased. The maximum bending angle can reach 39° under the actuation of SMA coils (Fig. S5B). In the twisting experiments, as depicted in Fig. 2E, the twisting angle curve was approximately linear, and the driving force curve was approximately parabolic. The maximum twisting angle was about 43° (Fig. S5C).

### Movement speed analysis

**Fig. 3.**
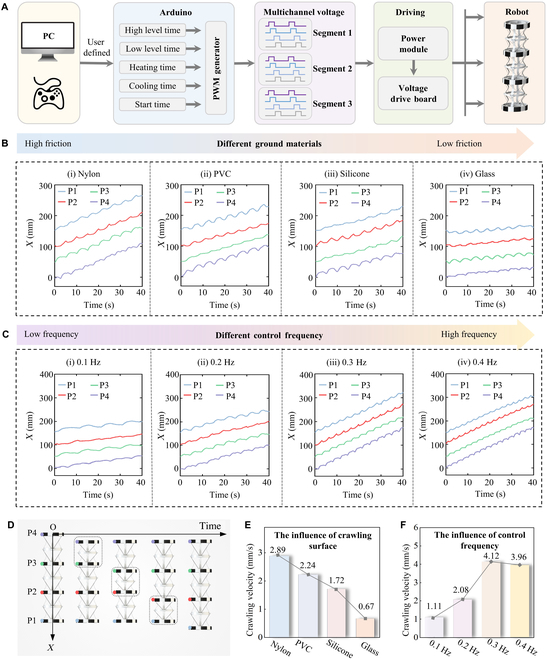
Movement speed analysis of the soft crawling robots inspired by *Drosophila* larvae. (A) Control block diagram of the soft crawling robots. (B) The robot’s movement displacement on diverse surfaces like nylon, PVC, silicone rubber, and glass. (C) The robot’s movement displacement under different control frequencies. (D) Schematic diagram of the robot’s movement displacement within a cycle. P1 to P4 are end points of the robot segments. (E) The average movement speeds of the soft crawling robot on different surfaces. (F) The average movement speeds of the soft crawling robot under different control frequencies.

Inspired by the locomotion of *Drosophila* larvae, the proposed robot moved in the form of a peristaltic locomotion. Each robot segment comprised 2 robot modules that deformed identically, with adjacent robot segments deforming sequentially over time. As illustrated in Fig. 3A and Fig. S6, the deformation of each segment needed to be controlled by 4 SMA coils, and the entire robot was controlled through 12 voltage channels. The voltage signals were pulse-width modulation (PWM) waves, including parameters such as high-level time, low-level time, heating time, cooling time, and start time (see Fig. S7). These parameters could be defined via a computer interface or manually adjusted using a handheld controller. The voltage signal output by the Arduino was power-amplified through a driver board before being connected to the SMA coils. The control signals and the friction between the robot and the movement surface significantly influenced the overall locomotive efficacy of the robot. This section addressed the robot’s movement speed across various surfaces and under different PWM cycle conditions.

Figure 3B shows the robot’s movement on diverse surfaces like nylon, polyvinyl chloride (PVC), silicone rubber, and glass, with each movement cycle lasting 5 s. It was observed that during its peristaltic advance, the robot experienced a certain amount of backward displacement during each cycle but still progressed forward at the end of the cycle. The robot’s locomotive efficiency varied with surface texture. The friction coefficients of different surfaces are plotted in Fig. S8. On rougher surfaces, such as nylon and PVC, it achieved more significant forward movement per cycle. The robot tended to experience slippage on smoother materials like glass, resulting in reduced speed. In addition, the robot’s movement speed at different cycle frequencies was analyzed. The PVC board was chosen as the movement surface, and the robot was controlled to move at frequencies of 0.1, 0.2, 0.3, and 0.4 Hz, as shown in Fig. 3C. It was observed that the robot’s speed increased with higher movement frequencies, but the forward displacement per cycle decreased. As the frequency continued to grow, the robot’s speed decreased, as its deformation could not keep up with the voltage changes.

Figure 3D and Fig. S9 detailed a schematic of the robot’s segment movement within a cycle. The robot began contracting from the last segment, sequentially moving forward until the peristaltic wave reached the first segment. P1 to P4 are end points of the robot segments, and the coordinate time curves of the 4 end points are obtained through video postprocessing software. Figure 3E quantified the robot’s average movement speeds on different surfaces. Notably, the robot achieved its highest average speed of 2.89 mm/s on the nylon surface. Figure 3F presented the average movement speeds of the robot at different frequencies. When operated at a 0.3-Hz cycle, the robot’s peak speed reached 4.12 mm/s.

### Multimodal motion experiment

**Fig. 4.**
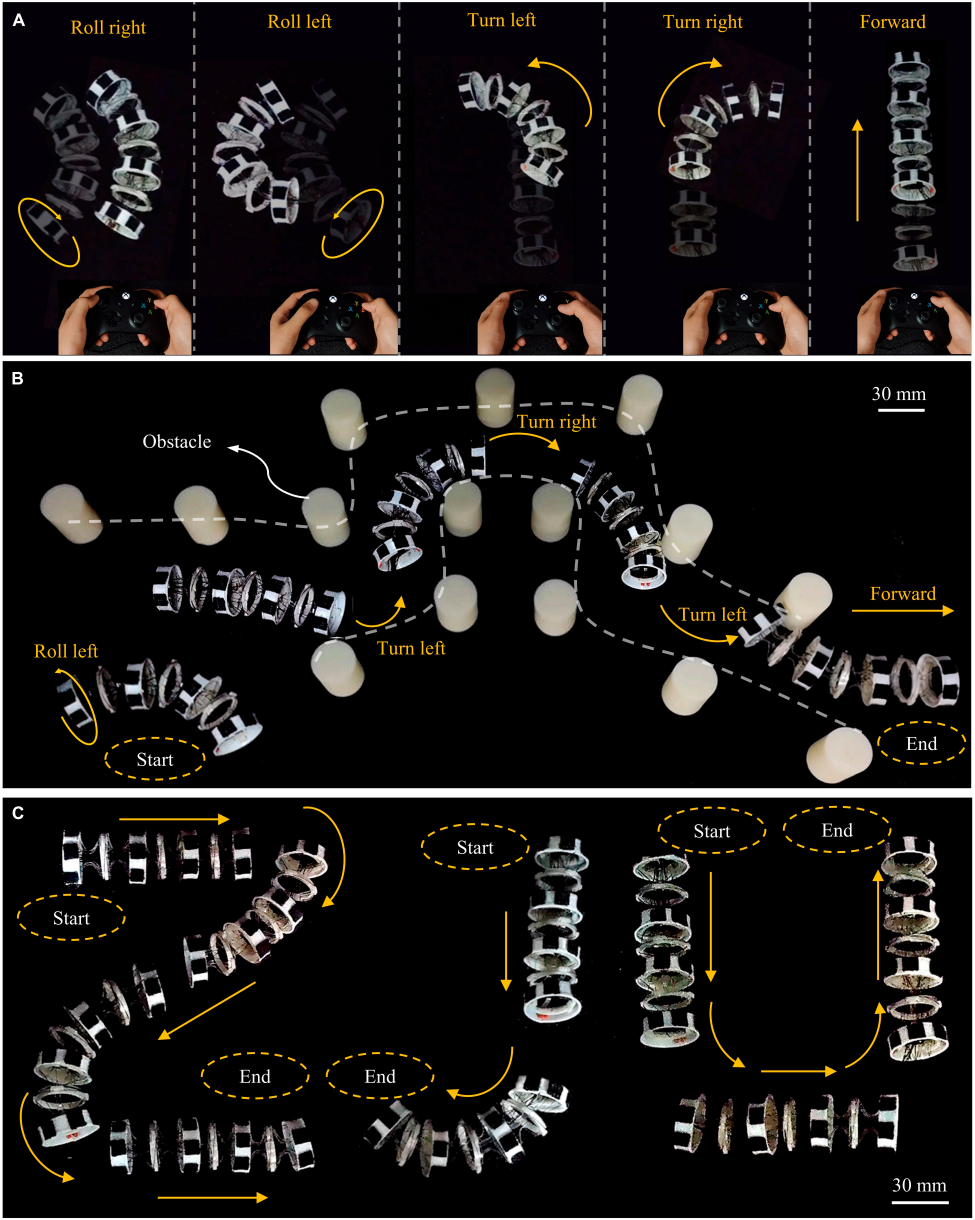
Multimodal motion experiment of the soft crawling robot inspired by *Drosophila* larvae. (A) Multimodal motion control of the soft crawling robot through the handle. (B) Obstacle avoidance experiment of the soft crawling robot. The robot was controlled in real time via a handle to adeptly navigate around the placed obstacles. The robot executed rolls, left turns, right turns, and forward movements, traversing narrow and winding paths, demonstrating its capability to explore complex environments. (C) Path tracking of “Z”-, “J”-, and “U”-shaped trajectories of the soft crawling robot. The robot was capable of effectively following the preset paths, although some deviations occurred during the turns.

In addition to crawling forward, this section highlights other significant locomotion modes of the robot. Different motion modes can be selected through the handle, enabling real-time switching of motion modes in response to the external environment, as depicted in Fig. 4A. The control strategies for various motion modes are detailed in Fig. S10 and Note S2.

Implementing the proposed motion strategy, we initially constructed a scenario with obstacles on a flat PVC board and controlled the robot to perform obstacle avoidance maneuvers, as illustrated in Fig. 4B. The robot, controlled in real-time via a handle, flexibly switched between different motion modes, adeptly navigating around the placed obstacles. It sequentially executed rolls, left turns, right turns, and forward movements, traversing narrow and winding paths, thereby demonstrating its capability to explore complex environments. It was observed that during movement, the robot made contact with obstacles. However, because of its soft body, such contact did not damage the robot.

Finally, we manually controlled the robot to trace “Z”-, “J”-, and “U”-shaped trajectories, as shown in Fig. 4C. The robot was capable of effectively following the preset paths, although some deviations occurred during the turns, with the robot exhibiting a tendency to follow an arc-like path while turning. These experiments showcased the proposed robot’s maneuverability and flexible movement capabilities.

### Pipeline exploration experiment

**Fig. 5.**
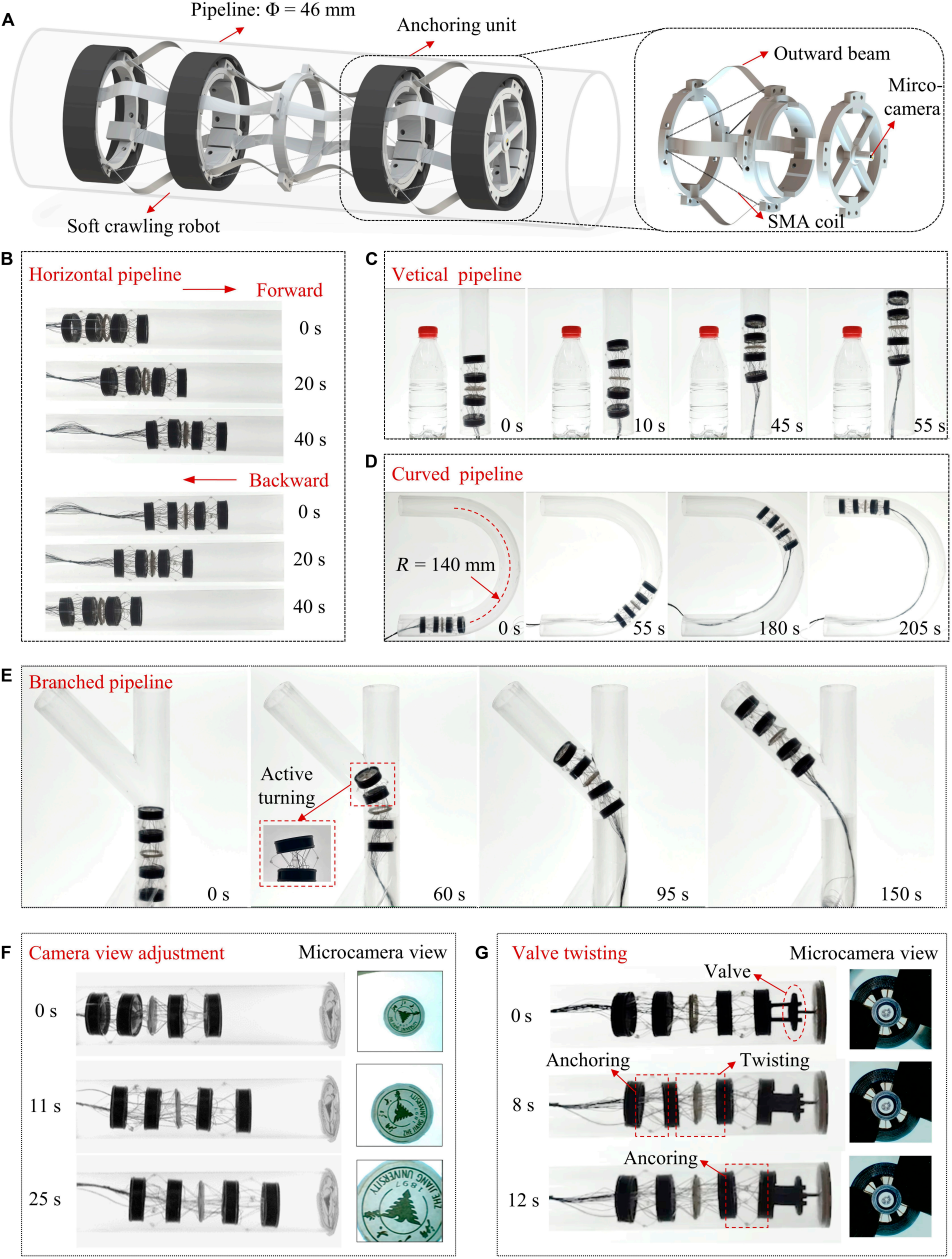
Pipeline exploration experiment of the soft crawling robot. (A) Structure diagram of the soft crawling robot used in the pipeline. The front and rear parts of the robot feature outward-bending support beams that can anchor onto the inner walls of the pipelines. (B) Crawling experiment in the straight horizontal pipeline. (C) Crawling experiment in the straight vertical pipeline. (D) Crawling experiment in the curved pipeline. (E) Crawling experiment in the branched pipeline. (F) Camera view adjustment experiment in the pipeline. (G) Valve rotation experiment in the pipeline.

The robot modules exhibit bistable characteristics, allowing the support beams to bend outward rather than just inward. This design enables the robot module to adjust its outer diameter and achieve active turning (as depicted in finite element method simulation results in Fig. S11), which greatly facilitates movement within pipelines. To validate this capability, we reconfigured the robot for pipeline exploration, as illustrated in Fig. 5A. The robot (weight, 10 g; length, 130 mm; diameter, 32 mm) is composed of 3 sections: The front and rear parts feature outward-bending support beams that can anchor onto the inner walls of the pipelines, while the middle section has support beams that bend inward. The small dark protrusions are made of rubber material and tied to the support beams to increase friction with the pipeline wall. In addition, a miniature camera is mounted on the robot’s head, serving to monitor the pipe environment.

Because of the high flexibility of the proposed robot, designing different control strategies enables it to perform multiple functions in pipelines (Fig. S12). When the robot crawls through a horizontal straight pipe, it begins by anchoring its head and then contracts the middle section, followed by anchoring the tail and unanchoring the head and middle section, achieving a forward crawling cycle. By reversing the anchoring sequence of the head and tail, the robot can crawl backward, as demonstrated in Fig. 5B. Moreover, when the straight pipe is positioned vertically, the same control strategy allows the robot to climb upward, counteracting its own weight, as illustrated in Fig. 5C. The robot successfully accomplishes this task, and the anchoring mechanism is effective, with no slippage occurring during the movement. To further demonstrate its versatility, the robot is placed inside a curved pipeline with a centerline radius of 140 mm, as depicted in Fig. 5D. Because of the robot’s inherent compliance, it can adapt to the pipeline environment and passively bend, allowing it to advance in curved pipes even when using control strategies designed for straight pipes. The robot’s ability to navigate branched pipelines was also tested, as shown in Fig. 5E. As the robot nears a junction, it initially steers into the desired branch by turning its head and bending the middle segment.

During pipeline inspection, adjusting the camera’s field of view is a critical requirement. To achieve this, the robot anchors its head to the pipeline and twists the middle segment, followed by anchoring the tail segment and then unanchoring the middle and head segments, resulting in the twisting of the robot’s head within the pipeline. As shown in Fig. 5F, as the robot twists, the pattern in the camera’s field of view rotates. In addition, the pattern enlarges as the robot moves forward. Furthermore, we demonstrated the robot’s valve-twisting capability, as shown in Fig. 5G. To our knowledge, this is the first time a soft robot achieves valve twisting in narrow pipelines. As depicted in Fig. S13, a fixture is initially installed on the robot’s head and inserted into the valve. Anchor the robot’s head and twist its middle section. Then, anchor the tail, unanchor the head, and twist the middle section in the opposite direction to complete one cycle of the twisting valve operation. The robot’s bidirectional twisting capability enables large-angle valve turning, verifying its operational capability in complex and narrow pipelines.

## Discussion

Inspired by the muscle structure and diverse movement patterns of *Drosophila* larvae, we developed a robot module based on SMA, which can achieve contracting, omnidirectional bending, and bidirectional twisting. Multiple modules can be assembled or detached through plug-and-play connectors. The modular soft crawler robot could be controlled via a handle, switching between modes like moving forward, turning, rolling, and twisting. It demonstrated flexible mode switching and obstacle avoidance in a narrow, confined maze and traced “Z”-, “J”-, and “U”-shaped paths. The modules’ bistable characteristic plays a pivotal role in this versatility, allowing the robot to conduct inspections and manipulations in varied pipe configurations, from horizontal and vertical to curved and branching ones, adjusting camera view and engaging in complex valve operations.

Soft crawling robots have recently gained significant research interest. We compared several representative soft crawlers, as shown in Table. A simple design involves linking single-degree-of-freedom actuators in series, advancing in the form of peristaltic waves [22,32]. More advanced iterations use dual parallel actuators, augmented with anchoring mechanisms such as electrostatic feet, enabling flexible forward motion and steering capabilities [1,33]. For even greater freedom of movement, a prevalent design incorporates 3 or more parallel actuators per module, enabling both stretching and omnidirectional bending. This configuration is particularly conducive to rolling by altering the robot’s center of mass. However, only a few robots demonstrate rolling due to the high demands on structure and actuator coordination [23]. Twisting motion, especially bidirectional twisting, is a scarcer feature in robot design, with only a few models able to twist in one direction, mainly inspired by the Kresling origami structure [34]. Implementing bidirectional twisting typically requires integrating multiple actuators.

**Table.** Comparison between the proposed robot and the other soft crawling robots

Ref	Actuation method	Robot design	Dimension (mm)	Weight (g)	Average forward speed (Bl/min)	Turning capability	Rolling capability	Twisting capability
[7]	Magnetic	Multilegged structure with flexible tapered feet	17 × 7	0.039	>240	**Y**	N	N
[34]	Magnetic	Four origami structure	Φ7.8 × 28	0.95	28.2	**Y**	N	**UT**
[33]	Pneumatic and electrostatic	Two parallel tube body with 2 electrostatic feet	60 × 30 × 135	43.4	7.2	**Y**	N	N
[23]	Pneumatic	Five vacuum-powered modules	Φ45 × 225	225	4.8	**Y**	**Y**	N
[22]	Pneumatic	Five peristaltic modules	Φ60 × 350	—	0.21	N	N	N
[1]	DEA	Two parallel DEAs with electrostatic feet	~125 × 20 × 85	~4	62.4	**Y**	N	N
[35]	DEA	Elongation and anchoring units	Φ47 × 6	2.2	71.4	N	N	N
[32]	Motor	Eight robot modules	Φ105 × 725	706	0.5	N	N	N
[36]	SMA	Omega-shaped structure	15 × 34 × 150	1.2	4	**Y**	N	N
[37]	SMA	Three 3-degree-of-freedom robot modules	Φ24 × 139	—	0.16	**Y**	N	N
**This work**	**SMA**	**Six 4-degree-of-freedom robot modules**	**Φ35 × 165**	**13**	**1.5**	**Y**	**Y**	**BT**

UT, unidirectional twisting; BT, bidirectional twisting.

In terms of actuator technology, magnetic-driven robots offer an ideal solution for miniaturization. Through careful engineering of the robot’s magnetization direction and manipulating the external magnetic field, multiple motion modes can be achieved [7]. However, the robot’s operational area is confined within the bounds of the external magnetic field, and the reliance on magnetic forces/torques results in a lower driving capability. Pneumatic robots are common because of simple integration but struggle with multimodal movement, often resulting in bulkier and heavier designs [22,23]. Dielectric elastomer actuator (DEA)-based robots move the fastest but require high-voltage drives to complicate their control systems, especially with multiple actuators for diverse movements [35]. Motor-driven robots offer the highest driving force but significantly increase system weight [32]. SMA coils are fine and lightweight with low driving voltage, making them particularly suitable for the design and integration of multimodal robots [36,37].

In general, our robot offers several advantages:

1. Advanced module structure: The robot modules are straightforward in design, easy to manufacture, and cost-effective. Utilizing an antagonistic arrangement of SMA, the robot modules can achieve contracting, omnidirectional bending, and bidirectional twisting. This marks a significant improvement over existing flexible robot module.
2. Diverse locomotion modes: The robot features a variety of movement modes, such as forward motion, turning, rolling, and twisting. Controlled by an external handheld device, these modes can be flexibly switched, significantly enhancing the robot’s multifunctionality.
3. Modular design approach: Modular design is particularly advantageous for repairing or replacing damaged modules and testing modules with new functionalities.
4. Reconfigurable architecture: The robot is designed with plug-and-play connectors between modules, enabling the robot to be easily reconfigured into different forms to suit various application scenarios and task requirements.
5. Bistable module characteristics: When the support beam of a module bends outward, it allows the module to increase its external diameter when compressed. This feature provides additional functionality, such as serving as an anchoring unit in pipelines. Combined with the twisting ability of the robot module, it enables diverse applications, especially first-time implementation of twisting valve within pipelines.

This robot demonstrates advanced movement and versatile application potential, paving the way for future soft crawling robot designs. However, the motion speed of SMA-driven robots is relatively slower, particularly those using a peristaltic gait due to sequential segment deformation. We are aiming to further improve the SMA’s thermal convection speed via active cooling methods, which would significantly increase the robot’s operational speed. In addition, advancements in microscale manufacturing could lead to further miniaturization of the robot, enabling it to operate in even more confined spaces, thus broadening the scope of its applicability.

## Materials and Methods

### Materials

The SMA coils used for actuating the proposed soft crawling robot is BMX150 (TOKI Corporation, Tokyo, Japan), with a wire diameter of 0.15 mm and an outer diameter of 0.6 mm. The connecting rings are made of heat-resistant Nylon glass fiber material through 3-dimensional (3D) printing, with a heat distortion temperature of 130 **°**C. The obstacles in the multimodal motion experiment are made of white resin material, and the pipelines in the pipeline exploration experiments are made of transparent resin material, all manufactured by 3D printing. The camera installed on the soft crawling robot is the OV6946 (OmniVision, California, USA), with 4 light-emitting diode lights and an overall outer diameter of 2 mm.

The handle used in the multimodal motion experiment is the Xbox 360 (Microsoft, Redmond, America). The Arduino model used for generating PWM signals is the Mega2560 (Centenary Electronics Co. Ltd., Shenzhen, China), which utilizes pins D2 (Digital Pin 2) to D13 to produce 12 PWM channels for driving the 3 segments of the robot. The board model used for power amplification of the PWM signals is AT8236 (Shenzhen Yahboom Technology Co. Ltd., Shenzhen, China), and the model of the power supply is MP3020D (Yan Jing Electronic Technology Co. Ltd., Suzhou, China), capable of generating an adjustable supply voltage ranging from 3.3 to 12 V. The filming equipment used in the experiments is a Huawei P30 (Huawei Technologies Co. Ltd., Shenzhen, China).

### Finite element analysis

The commercial finite element software ABAQUS is used for simulation analysis of the robot module. The upper and lower connecting rings are set as discrete rigid bodies, with their centroids set as the reference points. The lower connecting ring is subjected to a fixed constraint, while the tie constraint is applied between the contact surfaces of the support sheets and the connecting rings. The material properties of the support sheet are defined with Young’s modulus of 1,615 MPa and a Poisson’s ratio of 0.4. For compression conditions, the upper connecting ring is constrained to allow only axial displacement. Under bending conditions, the upper connecting ring is constrained to only have the bending degree of freedom. For torsional conditions, the upper connecting ring is permitted only axial displacement and axial torsional freedom.

## Data Availability

All data needed to evaluate the conclusions in the paper are present in the paper and/or the Supplementary Materials.

## Supplementary Materials

Supplementary 1 Note S1 and S2 Figs. S1 to S13 Movies S1 to S8
