# Supplementary material for: Drosophila Larvae-Inspired Soft Crawling Robot with Multimodal Locomotion and Versatile Applications
Source: Research (Wash D C). 2024 May 3;7:0357. doi: 10.34133/research.0357 (PMC11075670; doi:10.34133/research.0357)
Supplement: Supplementary 1 — Note S1 and S2 Figs. S1 to S13 Movies S1 to S8 [file research.0357.f1.zip › Supplementary Materials_clean.pdf]

Research

Supplementary Materials for

Drosophila Larvae-Inspired Soft Crawling Robot with Multi-Modal Locomotion and Versatile Applications

Qin Fang[1,2†], Jingyu Zhang[1,2†], Yinhui He[3], Nenggan Zheng[4], Yue Wang[1,2], Rong Xiong[1,2], Zhefeng Gong[3*], Haojian Lu[1,2,5*]

1 State Key Laboratory of Industrial Control and Technology, Zhejiang University, Hangzhou 310027, China
2 Institute of Cyber-Systems and Control, Zhejiang University, Hangzhou 310027, China
3 Medical School of Zhejiang University, Hangzhou 310027, China
4 College of Computer Science and Technology, Zhejiang University, Hangzhou 310027, China
5 Stomatology Hospital, School of Stomatology, Zhejiang University School of Medicine, Zhejiang Provincial Clinical Research Center for Oral Diseases, Key Laboratory of Oral Biomedical Research of Zhejiang Province, Cancer Center of Zhejiang University, Engineering Research Center of Oral Biomaterials and Devices of Zhejiang Province, Hangzhou 310000

†These authors contributed equally to this work.
*Address correspondence to: Haojian Lu; luhaojian@zju.edu.cn and Zhefeng Gong; zfgong@zju.edu.cn

This file includes:

Notes S1 to S2

Figures S1 to S13

Legends for Movies S1 to S8

Other Supplementary Materials for this manuscript include the following:

Movies S1 to S8 (.mp4 format)

Note S1. Kinematic model of the robot module

To describe the relationship between the multimodal deformation of the robot module and the lengths of four SMA coils, the kinematic model is established, as depicted in Fig. S3. The configuration parameters of the robot module are represented by four parameters: length $l \in (0, \infty)$, bending angle $\theta \in [-\pi, \pi]$, bending plane angle with respect to the X-axis $\varphi \in [0, 2\pi]$ and twisting angle $\delta \in [0, \pi/2]$. The lengths of the four SMA coils are denoted as l_1, l_2 and l_3, and l_4 respectively. a_1, b_1, c_1, and d_1 are the four endpoints on one side of SMA coils, while a_2, b_2, c_2, and d_2 are the endpoints on another side. The reference frame $\{o_b x_b y_b z_b\}$ is located on the bottom center of the robot module, while $\{o_t x_t y_t z_t\}$ and $\{\hat{o}_t \hat{x}_t \hat{y}_t \hat{z}_t\}$ are respectively the reference frames with and without twisting deformation locating on the top center.

The transformation matrix between the reference frame $\{o_b x_b y_b z_b\}$ and $\{o_t x_t y_t z_t\}$ can be represented as

$$\mathbf{T}_b^t = \mathbf{T}_c(l, \theta, \varphi)\mathbf{T}_z(\delta) \tag{1}$$

where $\mathbf{T}_c(l, \theta, \varphi)$ describes the bending and compression deformation, and $\mathbf{T}_z(\delta)$ describes the twisting deformation of the robot module. We have

$$\mathbf{T}_c(l, \theta, \varphi) = \begin{bmatrix} \mathbf{R}_c & \boldsymbol{p}_c \\ 0 & 1 \end{bmatrix} \tag{2}$$

By the constant curvature assumption, we have

$$\mathbf{T}_c(l, \theta, \varphi) = \begin{bmatrix} \cos^2\varphi(\cos\theta - 1) + 1 & \sin\varphi\cos\varphi(\cos\theta - 1) & \cos\varphi\sin\theta & l\cos\varphi(1 - \cos\theta)/\theta \\ \sin\varphi\cos\varphi(\cos\theta - 1) & \cos^2\varphi(1 - \cos\theta) + \cos\theta & \sin\varphi\sin\theta & l\sin\varphi(1 - \cos\theta)/\theta \\ -\cos\varphi\sin\theta & -\sin\varphi\sin\theta & \cos\theta & l\sin\theta/\theta \\ 0 & 0 & 0 & 1 \end{bmatrix} \tag{3}$$

And,

$$\mathbf{T}_z(\delta) = \begin{bmatrix} \cos\delta & -\sin\delta & 0 & 0 \\ \sin\delta & \cos\delta & 0 & 0 \\ 0 & 0 & 1 & 0 \\ 0 & 0 & 0 & 1 \end{bmatrix} \tag{4}$$

Thus, if configuration parameters $l, \theta, \varphi, \delta$ are given, $\mathbf{T}_b^t$ can be solved by the equation (3). By the CAD model of the robot module, we have: $\overrightarrow{o_b a_1} = (u, v, 0)$, $\overrightarrow{o_b b_1} = (-u, v, 0)$, $\overrightarrow{o_b c_1} = (-u, -v, 0)$ and $\overrightarrow{o_b d_1} = (u, -v, 0)$. Then, the position vectors $\overrightarrow{o_b a_2}$, $\overrightarrow{o_b b_2}$,$\overrightarrow{o_b c_2}$ and $\overrightarrow{o_b d_2}$ can

be solved by the following coordinate transformation.

$$[\overrightarrow{o_b a_2} \quad 1]^{\mathrm{T}} = \mathbf{T}_b^t \cdot \mathbf{T}_z(-\tfrac{\pi}{2}) \cdot [\overrightarrow{o_b b_1} \quad 1]^{\mathrm{T}} \tag{5}$$

$$[\overrightarrow{o_b b_2} \quad 1]^{\mathrm{T}} = \mathbf{T}_b^t \cdot \mathbf{T}_z(-\tfrac{\pi}{2}) \cdot [\overrightarrow{o_b c_1} \quad 1]^{\mathrm{T}} \tag{6}$$

$$[\overrightarrow{o_b c_2} \quad 1]^{\mathrm{T}} = \mathbf{T}_b^t \cdot \mathbf{T}_z(-\tfrac{\pi}{2}) \cdot [\overrightarrow{o_b d_1} \quad 1]^{\mathrm{T}} \tag{7}$$

$$[\overrightarrow{o_b d_2} \quad 1]^{\mathrm{T}} = \mathbf{T}_b^t \cdot \mathbf{T}_z(-\tfrac{\pi}{2}) \cdot [\overrightarrow{o_b a_1} \quad 1]^{\mathrm{T}} \tag{8}$$

where

$$\mathbf{T}_z\left(-\frac{\pi}{2}\right) = \begin{bmatrix} 0 & 1 & 0 & 0 \\ -1 & 0 & 0 & 0 \\ 0 & 0 & 1 & 0 \\ 0 & 0 & 0 & 1 \end{bmatrix}$$

The lengths of four SMA coils can be represented as

$$l_1 = |\overrightarrow{a_1 a_2}| = |\overrightarrow{o_b a_2} - \overrightarrow{o_b a_1}| \tag{9}$$

$$l_2 = |\overrightarrow{b_1 b_2}| = |\overrightarrow{o_b b_2} - \overrightarrow{o_b b_1}| \tag{10}$$

$$l_3 = |\overrightarrow{c_1 c_2}| = |\overrightarrow{o_b c_2} - \overrightarrow{o_b c_1}| \tag{11}$$

$$l_4 = |\overrightarrow{d_1 d_2}| = |\overrightarrow{o_b d_2} - \overrightarrow{o_b d_1}| \tag{12}$$

If configuration parameters $l, \theta, \varphi, \delta$ are given, the lengths of SMA coils can be solved by equation (9)-(12), and vice versa.

Note S2. Control voltage parameters during multimodal movement.

During obstacle avoidance experiments, the frequency of the robot control voltage is set at 0.2 Hz, with a maximum voltage of 5V and a minimum of 0V. The handle allows real-time control over the robot's motion modes, including forward movement, left and right turns, and rolling, to navigate obstacles flexibly. Thus, the control parameters of the voltage change with different motion modes.

For straight movement, as depicted in Fig. S7, the parameters for the 12 groups of SMA control voltage are as follows: high level time is 25ms, low level time is 25ms, heating time is 2.5s, and cooling time is 2.5s. The start time is dependent on the robot's segments, with the same segment's four SMAs having identical start times. The first segment's start time is 0; the second segment's start time is 2.5 s; the third segment's start time is 5s.

During turns, the contraction level of the SMA on the bending side is increased, with a high-level time of 30ms and a low-level time of 20ms, while the heating and cooling times remain at 2.5s. Conversely, the contraction level of the SMA on the opposite side is decreased, with a high-level time of 20ms and a low-level time of 30ms, and identical heating and cooling times. The start times for different segments remain the same as those during straight movement.

When rolling, one side of the robot begins to bend first, with the SMA on this side having a high-level time of 30ms and a low-level time of 20ms, and heating and cooling times of 2.5s. Then, the SMA on this side is powered off, and the adjacent side's SMA bends. This process continues around the robot's circumference, with the start times of the four SMAs in the same segment differing, and the start times of the control voltages for the adjacent bending sides along the segment circumference successively differing by 2.5s.

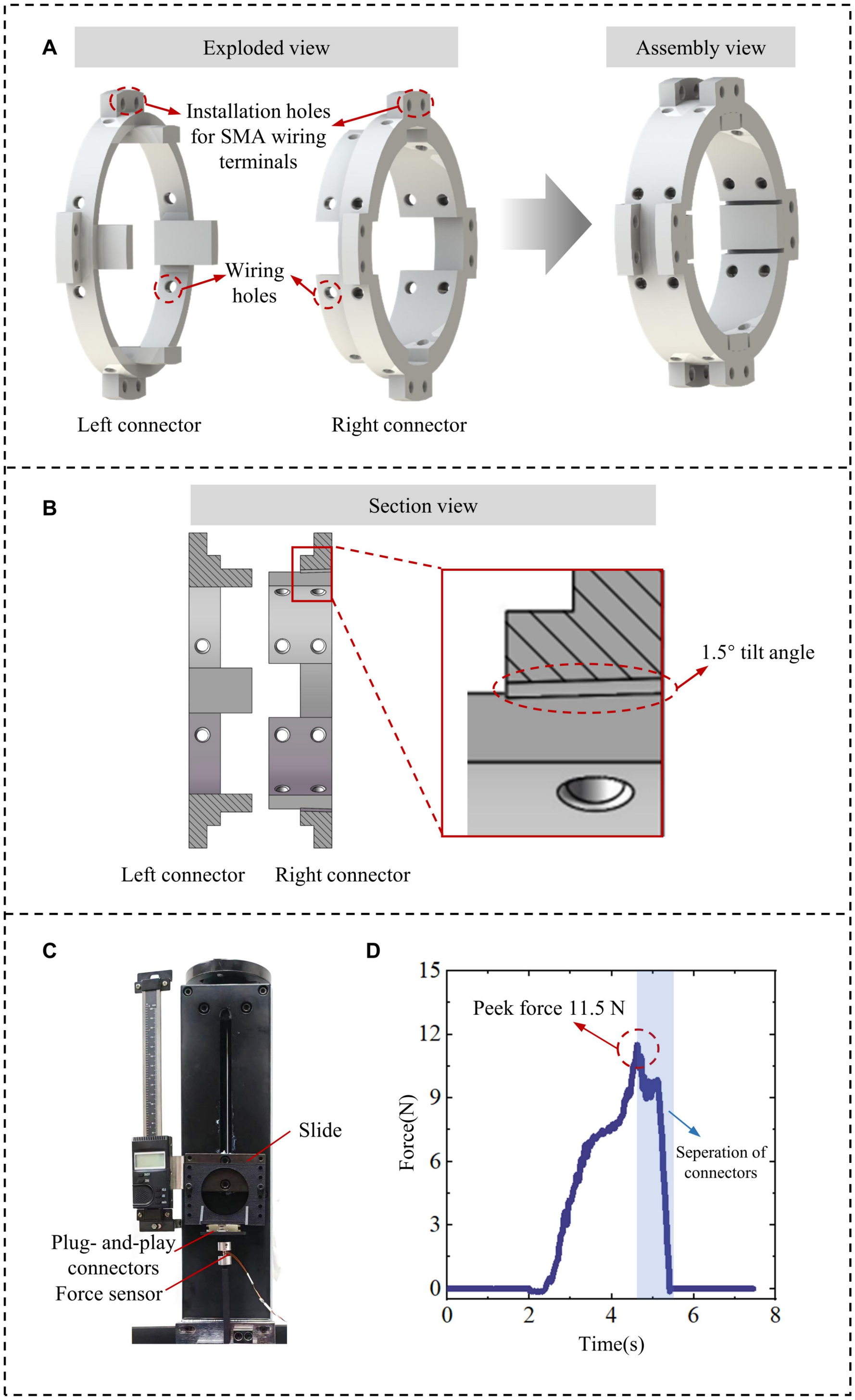

Fig. S1. Schematic diagram of connector structure and reliability testing experiment. (A) Explosion view and assembly view of the connector structure. (B) The sectional view of the connector structure. There is a tilt angle of 1.5° at both ends of the modules, enabling the robot modules to be quickly connected and disassembled through an interference fit. (C) Test scenario for tensile force required for separate connectors. (D) The tensile force curve obtained from the connector separation test. To separate each pair of connectors, a force of 11.5 N is needed, which is 88 times the weight of the robot itself.

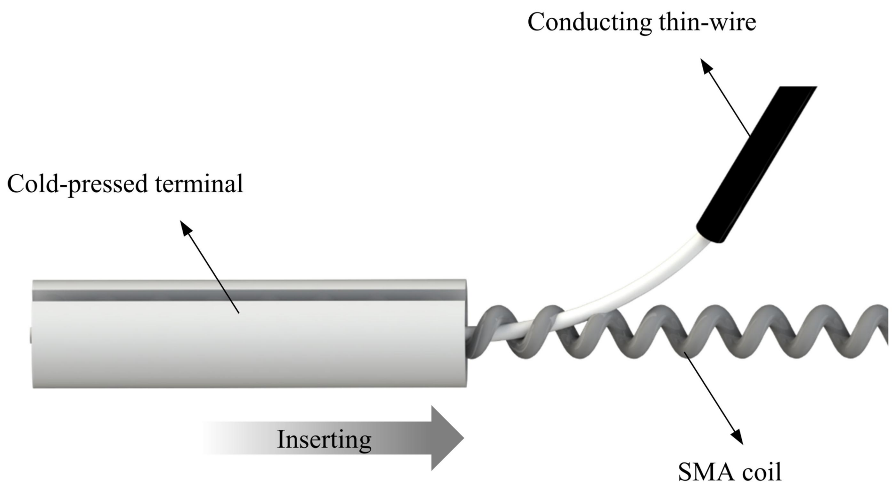

Fig. S2. Installation diagram of SMA coils. The cold-pressed terminal is used to connect the SMA coil to the conducting thin-wire.

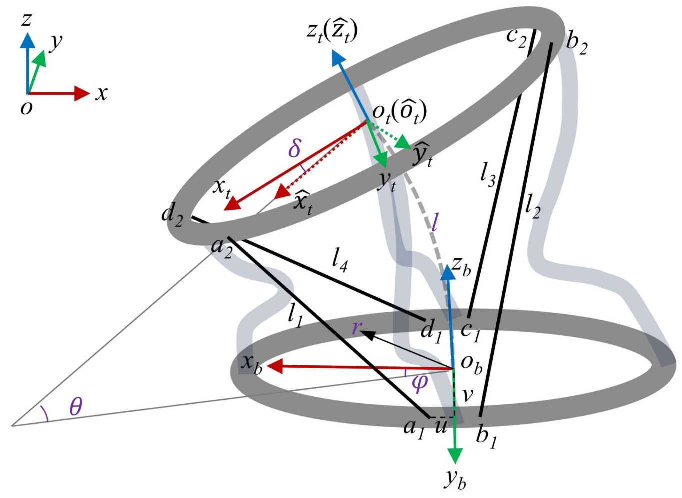

Fig. S3. Kinematic model diagram of robot module actuated by SMA coils. a_1, b_1, c_1 and d_1 are one of the vertices of four SMA coils, while a_2, b_2, c_2 and d_2 are another of the vertices of four SMA coils. l_1, l_2 and l_3, and l_4 are the lengths of the four SMA coils.

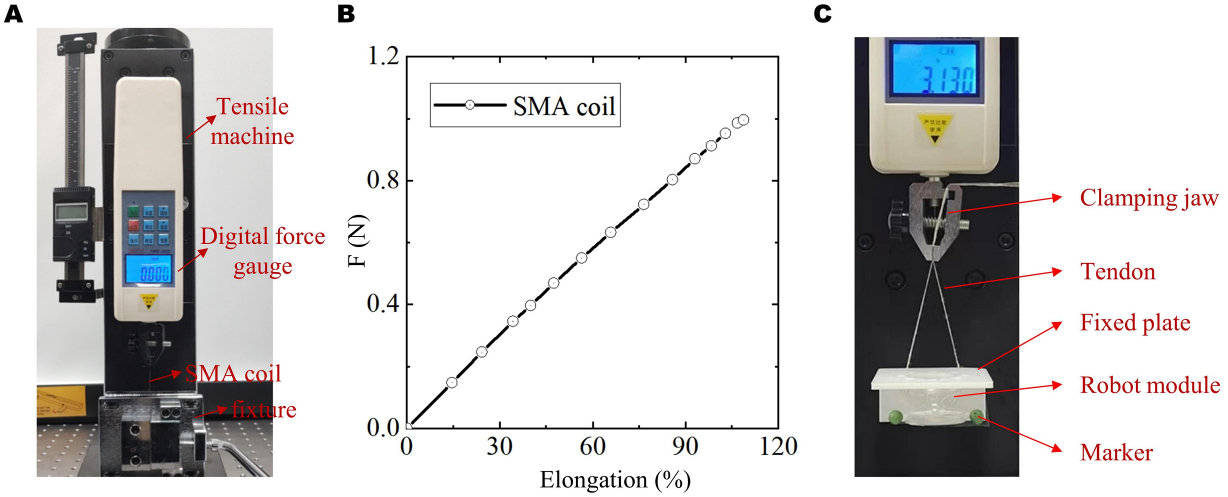

Fig. S4. Characterization of SMA coil and the robot module. (A) Characterization experimental setup for SMA coil. The SMA coils is respectively held by the fixture of the tensile machine and digital force gauge. **(B)** Force-elongation curve of SMA coil. With the increase of elongation, the force increased linearly. **(C)** Characterization experiments of the robot module. The robot module was mounted on the tensile machine, and the clamping jaw pulling the tendon to simulate the contraction of the SMA coil.

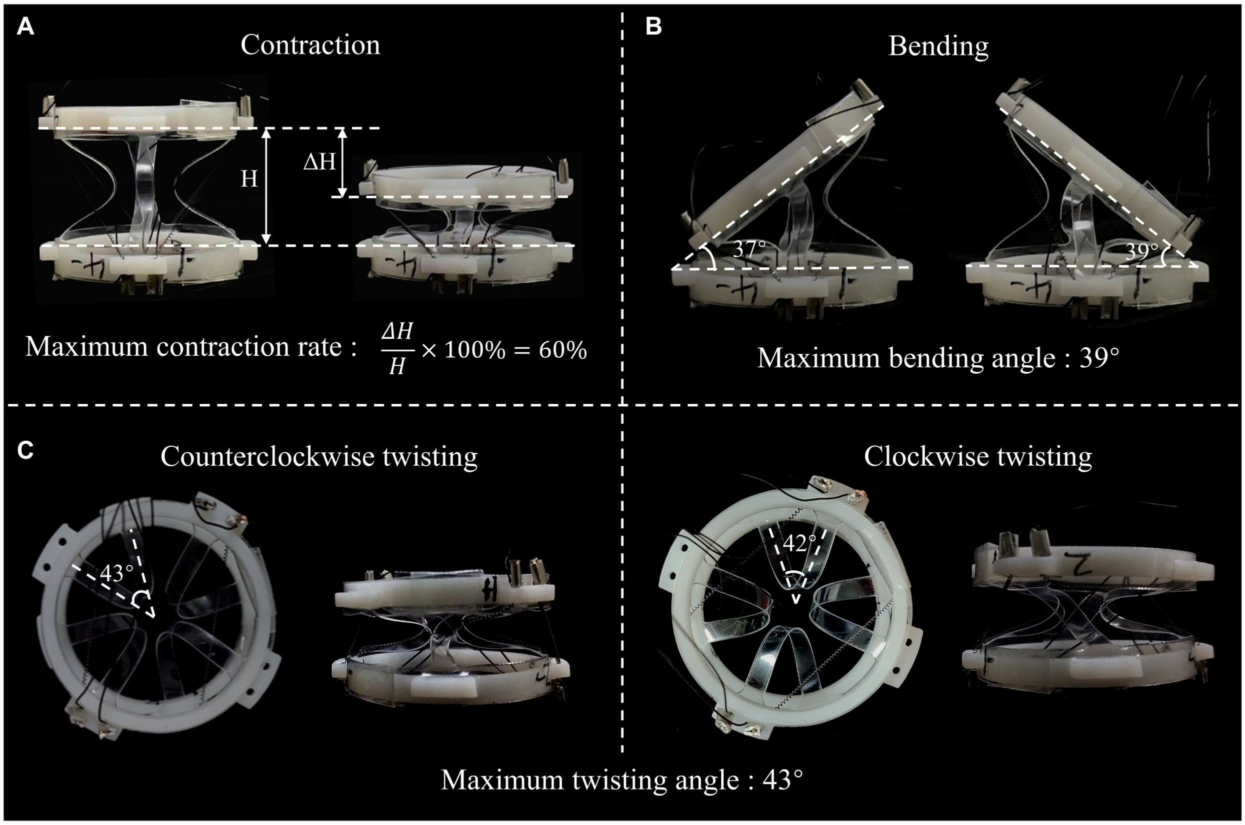

Fig. S5. Snapshots of the multimodal deformation of the robot module. (A) Contracting deformation of the robot module. When the four SMA coils are applied with the same control voltages, the robot module will contract axially from the initial state. The maximum contraction rate is 60%. **(B)** Bending deformation of the robot module. When the two adjacent SMA coils are applied with the same control voltages, the robot module will bend. By switching the adjacent SMA coils, the robot can bend in any direction. The maximum bending angle is 39°. **(C)** Twisting deformation of the robot module. When the two opposite SMA coils are applied with the same control voltages, the robot module will twist. By switching the opposite SMA coils, the robot can twist in clockwise or counterclockwise direction. The maximum twisting angle is 43°.

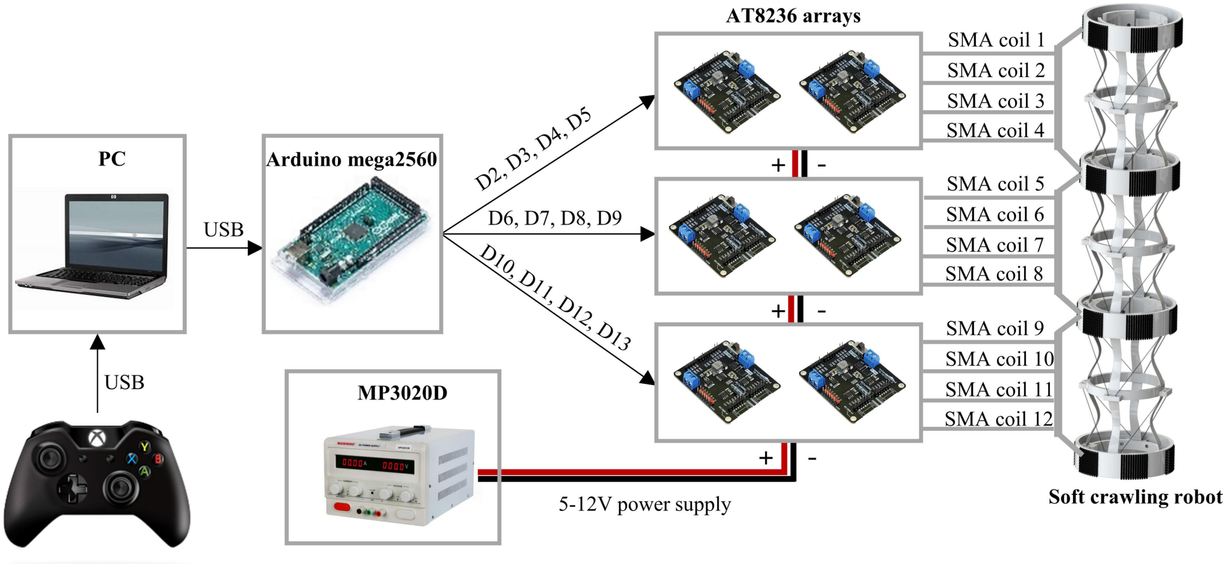

Fig. S6. Electrical control system for the proposed soft crawling robot. The control system consists of a personal computer (PC) for high-level motion control, an Arduino mega2560 using D2-D13 to generate PWM signals, AT8236 arrays used for amplifying the PWM signals, a MP3020D used for power supply of AT8236 arrays.

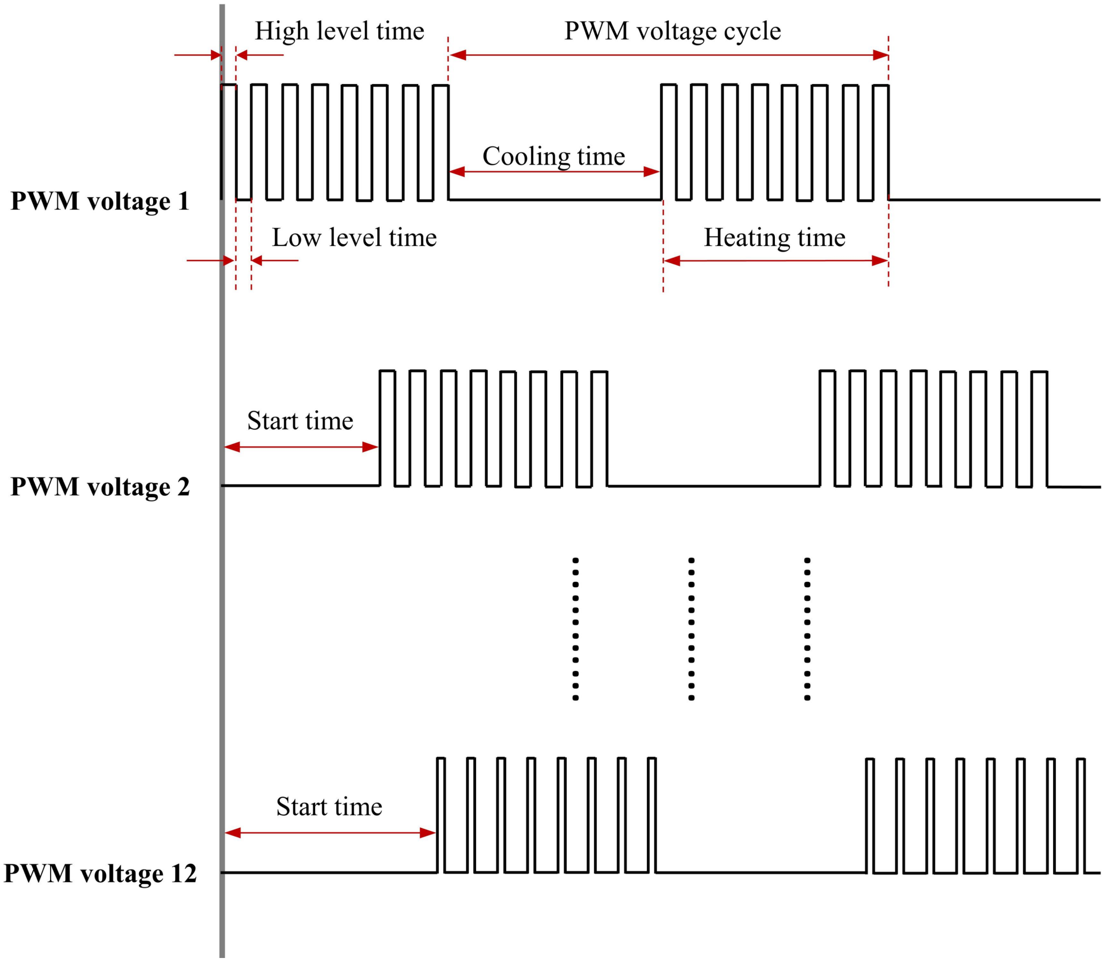

Fig. S7. PWM control voltage of 12 sets of SMA coils. The voltage control signal for each SMA includes start time, high-level time, low-level time, heating time, and cooling time. By properly adjusting these parameters, the robot can generate various modes of motion.

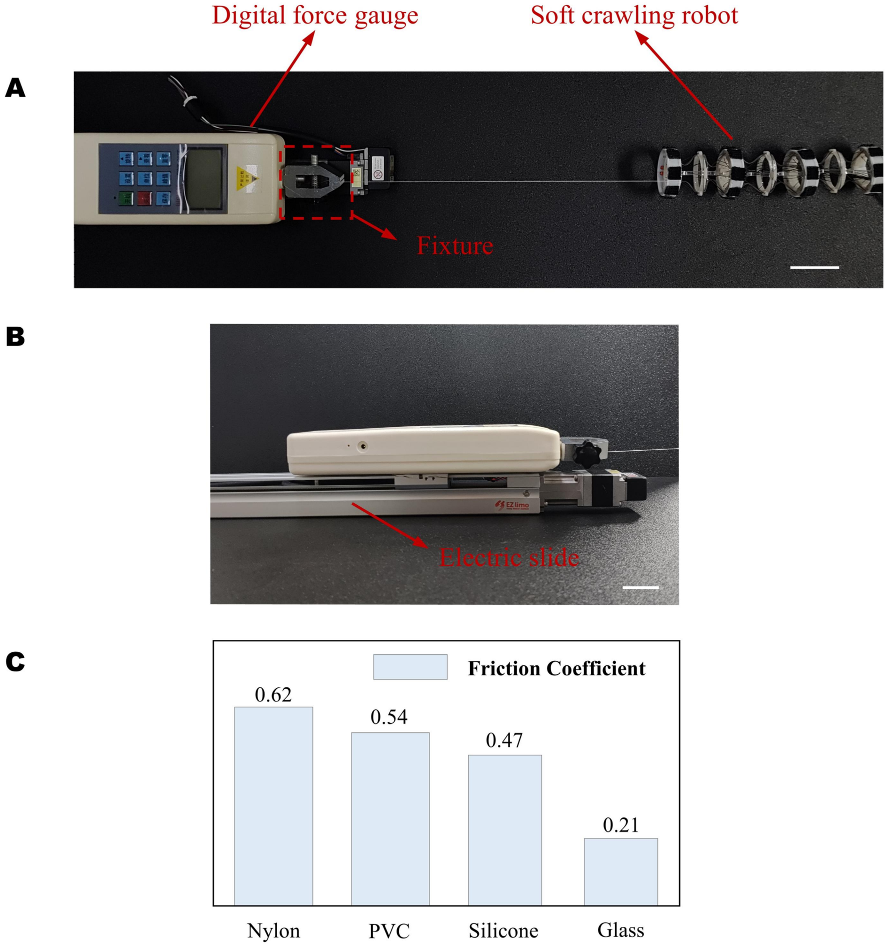

Fig. S8. Friction coefficient measurement on different surfaces. The digital force gauge is fixed on the electric slide, connected to the soft crawling robot via an inextensible soft thread. The electric slide moves at a speed of 1mm/s, pulling the robot across various material surfaces. The force displayed on the digital force gauge represents the robot's frictional force. Dividing this frictional force by the robot's gravity to obtain the friction coefficient. **(A)** Top view of experimental setup for friction coefficient measurement. Scale bar: 35 mm. **(B)** Side view of experimental setup for friction coefficient measurement. Scale bar: 35 mm. **(C)** Measured friction coefficients on different surfaces.

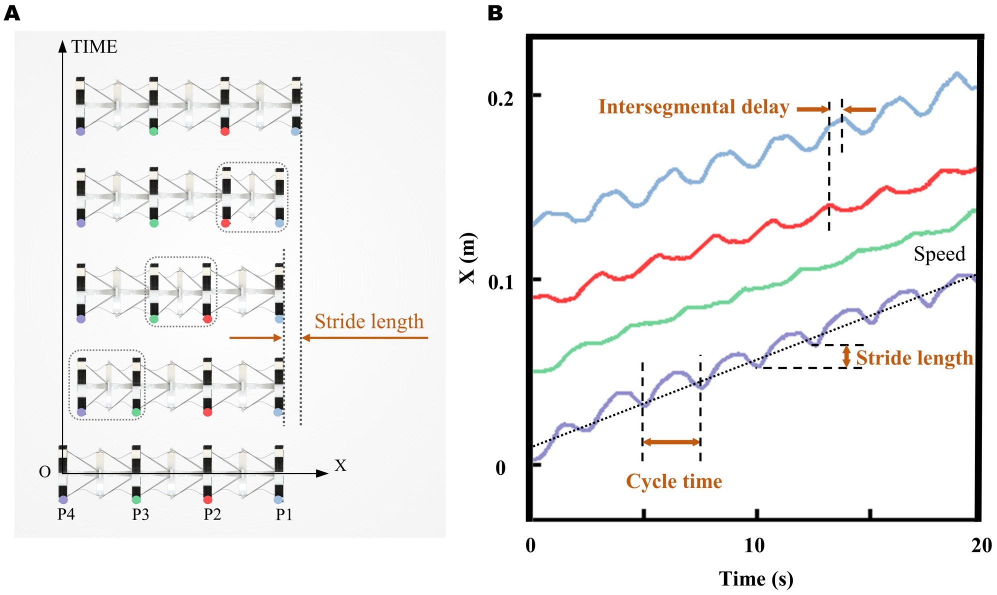

Fig. S9. Forward crawling motion analysis of the soft crawling robot. (A) The motion diagram of each segment of the crawling robot during forward crawling. P_1, P_2, P_3 and P_4 are the four positioning markers. **(B)** The analysis of the robot's movement. Based on the robot's motion curve, the cycle time, stride length, the robot' average speed, and the delay between different segments can be obtained.

Motion mode	Schematic diagram	Control strategy	Description
Crawling forward	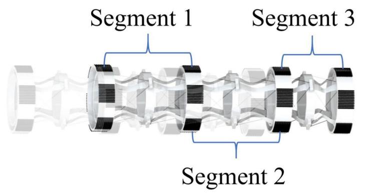	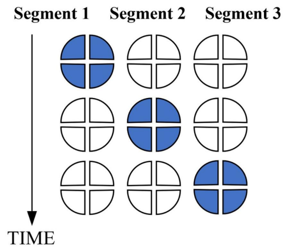	The soft crawling robot contracts from segment 1 to segment 3, and there is phase delay between adjacent segments.
Turning	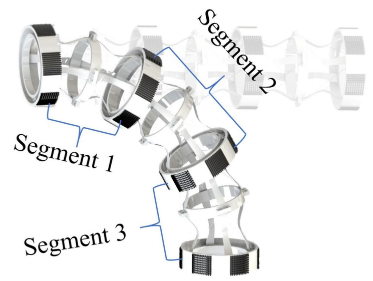	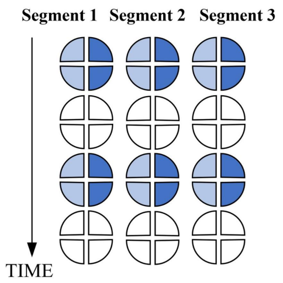	Each segment of the soft crawling robot contracts and bends, then elongates when the power is cut off. By repeating this cycle, the robot is able to move forward while simultaneously turning.
Rolling	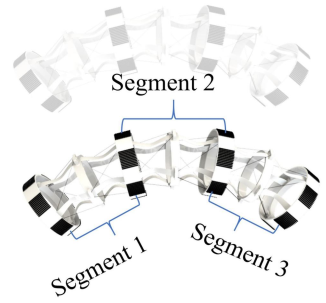	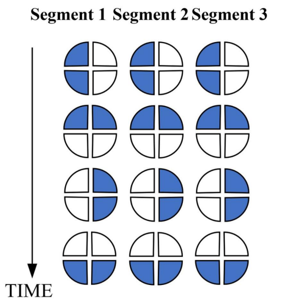	Each segment of the soft crawling robot bend in one direction and switch the bending direction circumferentially. By changing the switching direction, the robot can achieve rolling to the left or right.
Twisting	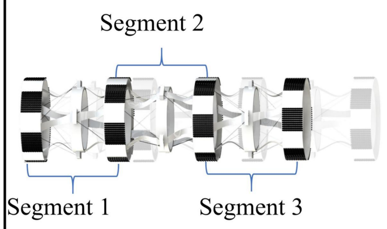	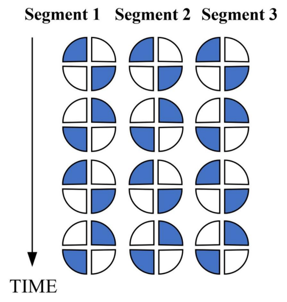	Each segment of the soft crawling is twisted in a counterclockwise direction and then clockwise direction.

Fig. S10. Multimodal motion control strategy for the ground crawling robot. Each segment has four actuators, where blue signifies activation, with darker blue indicating higher activation levers, and white means no activation.

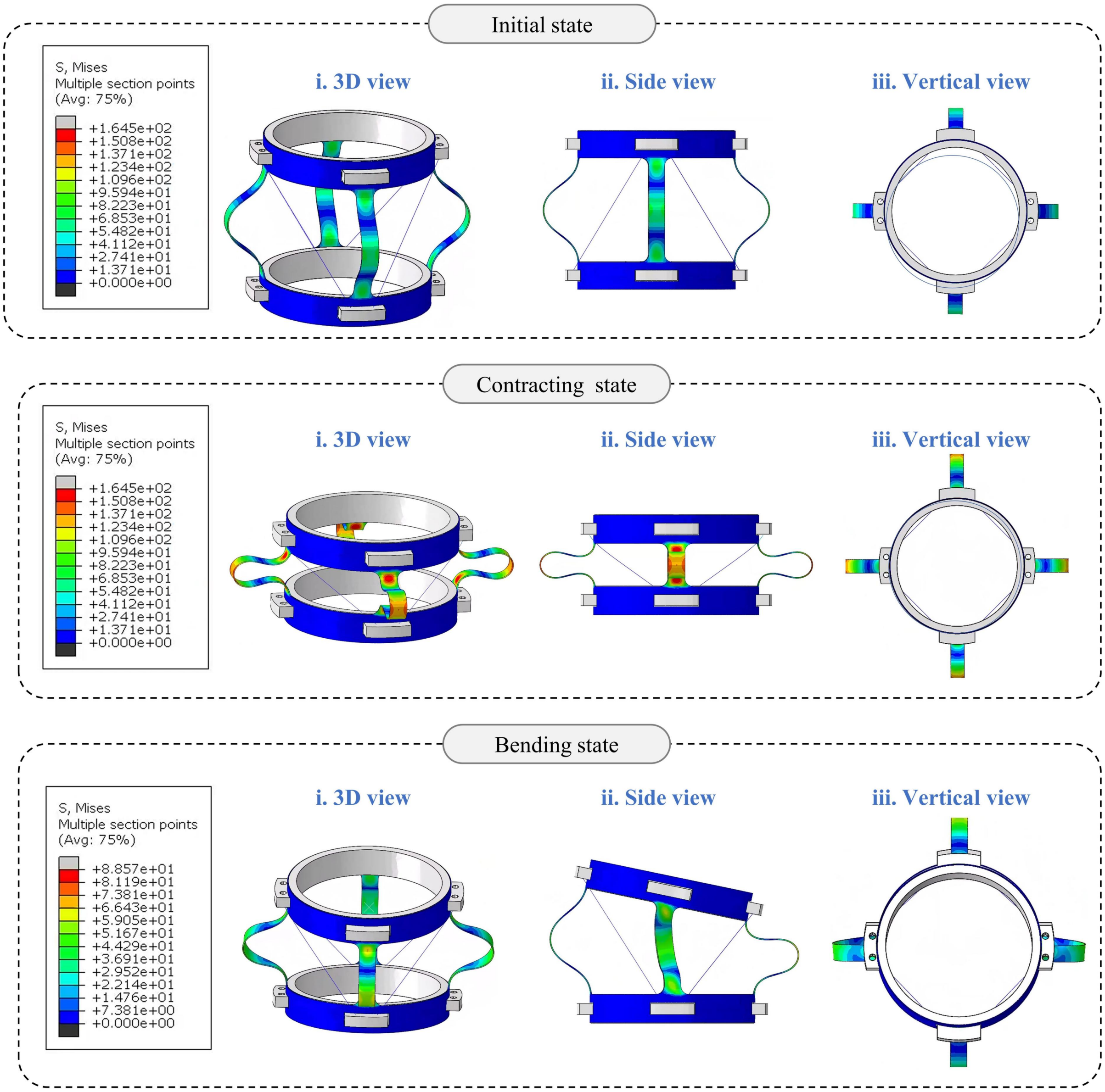

Fig. S11. FEA analysis of the robot module with the support beams bending outwards. (A) The initial state of the robot module. i, ii and iii respectively show the 3D view, side view and vertical view of the simulation model. **(B)** The contracting state of the robot module. i, ii and iii respectively show the 3D view, side view and vertical view of the simulation model. **(C)** The bending state of the robot module. i, ii and iii respectively show the 3D view, side view and vertical view of the simulation model.

Motion mode	Schematic diagram	Control strategy	Description
Crawling forward	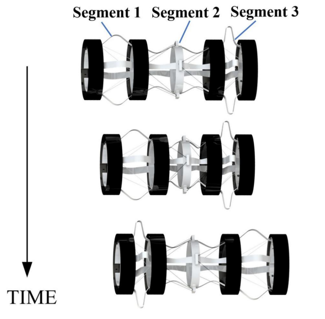	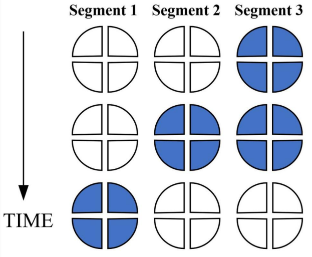	The robot's motion cycle begins with anchoring the its head, then contracts the middle section, followed by anchoring the tail and relaxing the head andmiddle section, achieving a forward crawling cycle. By reversing the anchoring sequence of the head and tail, the robot can crawl backwards
Turning	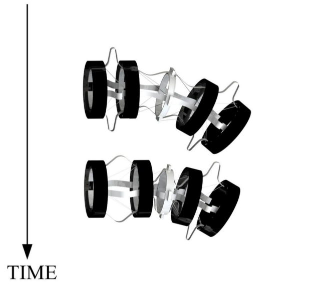	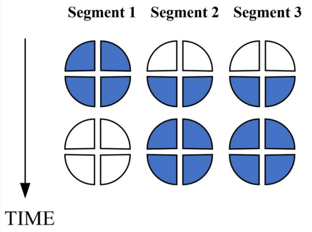	During every motion cycle, the segment 1 anchors itself within the pipeline, and segment 2 and 3bend towards the target direction. Following this, the segment 2 and 3 contracts while the segment 1 is released.
Twisting (Camera view adjustment)	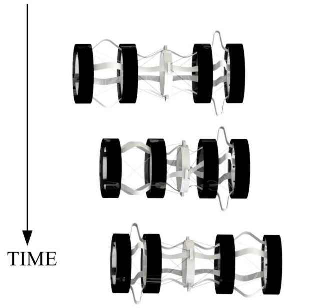	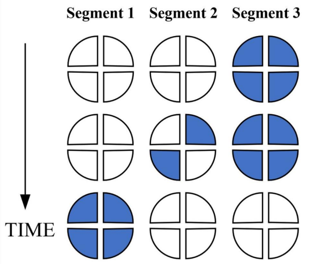	During every motion cycle, segment 3 firstly contract, then segment 2 twist in a clockwise or counterclockwise direction. Finally, segment 1 contacts to anchor the pipeline, and segment 2 and 3 are released. .
Twisting (Valve rotation)	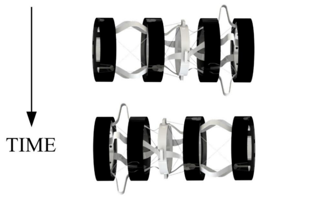	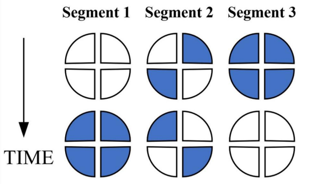	Under every motion cycle, segment 3 firstly contracts and segment 2 twists toward one direction. Next, segment 1 contracts to anchor the pipeline and segment 2 twists toward another direction .

Fig. S12. Multimodal motion control strategy for the pipeline crawling robot. Each segment has four actuators, where blue signifies activation, and white means no activation.

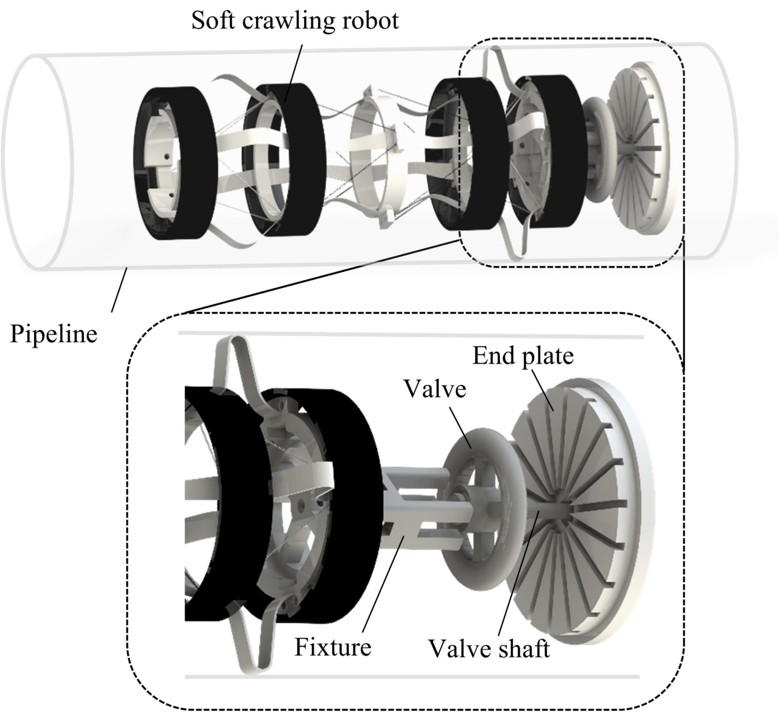

Fig. S13. Structure diagram of the valve rotation mechanism. The soft crawling robot uses the fixture to connect the valve that installed on the end plate by the valve shaft. The valve can be rotated around the valve shaft. During experiment, the soft crawling robot rotates the valve relying on its twisting ability.

Legends for Movies S1 to S8

Movie S1. Teleoperation Control of Multimodal Locomotion. The soft crawling robot is controlled by the handle to crawl forward, turn left, turn right, roll left and roll right, respectively.

Movie S2. Obstacle Avoidance experiment in unstructured environment. The soft crawling robot is controlled to traverse a man-made unstructured environment relying on its multimodal motion capability.

Movie S3. Path Tracking Experiment. The soft crawling robot is controlled to crawl along preset Z-shaped, J-shaped and U-shaped path.

Movie S4. Crawling Experiment in Horizontal Pipeline. The soft crawling robot is controlled to crawl forward and backward in a horizontal pipeline.

Movie S5. Crawling Experiments in Vertical and Curved Pipelines. The soft crawling robot is controlled to crawl in a vertical and a curved pipeline, respectively.

Movie S6. Active Steering Experiment in Branched Pipeline. The soft crawling robot is controlled to crawl in a branched pipeline. At the junction, the robot can actively steer into the designated pipeline.

Movie S7. Camera View Adjustment Experiment in Pipeline. The soft crawling robot is controlled to twist the body inside the pipeline to adjust the camera's view.

Movie S8. Valve Rotation Experiment in Pipeline. The robot soft crawling robot is controlled to rotate the valve inside the pipeline to show the application capability.
